# Regional expression of Pax7 in the brain of *Xenopus laevis* during embryonic and larval development

**DOI:** 10.3389/fnana.2013.00048

**Published:** 2013-12-24

**Authors:** Sandra Bandín, Ruth Morona, Nerea Moreno, Agustín González

**Affiliations:** Department of Cell Biology, Faculty of Biology, University ComplutenseMadrid, Spain

**Keywords:** Pax genes, immunohistochemistry, segmental organization, diencephalon, mesencephalon, brain evolution

## Abstract

Pax7 is a member of the highly conserved Pax gene family that is expressed in restricted zones of the central nervous system (CNS) during development, being involved in early brain regionalization and the maintenance of the regional identity. Using sensitive immunohistochemical techniques we have analyzed the spatiotemporal pattern of Pax7 expression in the brain of the anuran amphibian *Xenopus laevis*, during development. Pax7 expression was first detected in early embryos in the basal plate of prosomere 3, roof and alar plates of prosomere 1 and mesencephalon, and the alar plate of rhombomere 1. As development proceeded, Pax7 cells were observed in the hypothalamus close to the catecholaminergic population of the mammillary region. In the diencephalon, Pax7 was intensely expressed in a portion of the basal plate of prosomere 3, in the roof plate and in scattered cells of the thalamus in prosomere 2, throughout the roof of prosomere 1, and in the commissural and juxtacommissural domains of the pretectum. In the mesencephalon, Pax7 cells were localized in the optic tectum and, to a lesser extent, in the torus semicircularis. The rostral portion of the alar part of rhombomere 1, including the ventricular layer of the cerebellum, expressed Pax7 and, gradually, some of these dorsal cells were observed to populate ventrally the interpeduncular nucleus and the isthmus (rhombomere 0). Additionally, Pax7 positive cells were found in the ventricular zone of the ventral part of the alar plate along the rhombencephalon and the spinal cord. The findings show that the strongly conserved features of Pax7 expression through development shared by amniote vertebrates are also present in the anamniote amphibians as a common characteristic of the brain organization of tetrapods.

## Introduction

The development of the central nervous system (CNS) depends on a number of multileveled interactions of products of several large gene families, which act as transcriptional regulators and signaling molecules that guide molecular events in the regional specification, cellular determination and, ultimately, morphohistogenesis. Thus, morphological similarities or differences during vertebrate evolution are determined by the expression of pivotal genes, and related species frequently show common patterns of expression of developmental genes in specific regions of the CNS, supporting their homology (Davidson, 2006; Davidson and Erwin, 2006; Carroll, 2008).

Pax genes encode a family of highly conserved transcription factors characterized by the presence of a paired domain that confers sequence-specific binding to DNA; in addition, Pax transcription factors may also have an octapeptide motif and part or all of a homeobox DNA-binding domain (Balczarek et al., 1997; Chi and Epstein, 2002; Vorobyov and Horst, 2006; Lang et al., 2007; Wang et al., 2010). Among the Pax genes, Pax7 has the paired domain, the octapeptide motif, and the homeobox domain and is expressed in especific regions of the developing brain. It is involved in neuronal proliferation, brain regionalization, cell differentiation and neuronal survival (Wehr and Gruss, 1996; Lang et al., 2007; Thompson et al., 2008; Wang et al., 2008; Thompson and Ziman, 2011).

Previous research has established that the gene Pax7 is expressed during early brain development in regionally restricted patterns (highly overlapping with Pax3) within the diencephalon, mesencephalon, hindbrain and spinal cord in all species studied (Goulding and Paquette, 1994; Mansouri et al., 1996; Seo et al., 1998; Borycki et al., 1999; Minchin and Hughes, 2008; Thompson et al., 2008; Joven et al., 2013a,b) where it interacts with several additional genes as Fgf8, En2, Pax2-5 (Matsunaga et al., 2001), Pax3 (Seo et al., 1998; Thompson et al., 2008; Maczkowiak et al., 2010; Agoston et al., 2012), and Pax6 (Nomura et al., 1998; Thompson et al., 2007). The mutation of Pax genes are linked to diseases or physical defects (e.g., aniridia). Such profound effects confirm Pax proteins as “master” controllers (Gehring, 1996; Underhill, 2000) and essential morphoregulators during development (Tremblay and Gruss, 1994).

Most anatomical data about Pax7 expression in the developing brain were obtained in amniotes but important data have also been reported in fishes (Seo et al., 1998; Sibthorpe et al., 2006) and urodele amphibians (Joven et al., 2013a,b), highlighting conserved features and also interesting differences between amniotes and anamniotes. Fragmentary data are also available about the expression of this transcription factor in particular zones of the brain of *Xenopus laevis* (Ziman et al., 2001; Maczkowiak et al., 2010; Morona et al., 2011; Domínguez et al., 2013b). However, given the importance of this anuran species as a model of brain development in anamniotes, we have conducted a comprehensive study of the spatiotemporal distribution of the Pax7-immunoreactive cells (Pax7 cells) throughout the embryonic and larval brain development of *Xenopus laevis.* The analysis of the results has been made in the context of current neuromeric model of the brain that facilitate comparisons across vertebrate species and serve to assess evolutionary trends (Gilland and Baker, 1993; Marín and Puelles, 1995; Puelles et al., 1996; Fritzsch, 1998; Cambronero and Puelles, 2000; Díaz et al., 2000; Puelles and Rubenstein, 2003; Straka et al., 2006). The immunohistochemical techniques employed in the present study allow high-resolution analysis of expressing cells (Hitchcock et al., 1996; Wullimann and Rink, 2001; González and Northcutt, 2009; Domínguez et al., 2011, 2013a,b; Ferreiro-Galve et al., 2012; Joven et al., 2013a,b). To identify accurately the cell groups expressing Pax7, we used combined immunohistofluorescence to simultaneously reveal several neuronal markers, which served to highlight the boundaries and landmarks of numerous brain regions, as previously reported (González et al., 1994, 2002; Tuinhof et al., 1994; Barale et al., 1996; López and González, 2002; Morona and González, 2008, 2009, 2013; Domínguez et al., 2013a,b). These markers included the γ-amino butyric acid (GABA), calretinin (CR), nitric oxide synthase (NOS), tyrosine hydroxylase (TH), and the transcription factors Nkx2.1 and Otp. This study shows an extremely conserved distribution pattern of Pax7 cells between amphibians and other vertebrates. In addition, these experiments helped to clarify the actual position of many cell groups, to identify distinct boundaries, and to follow the relative position of developing cell subpopulations in the brain of *Xenopus*, which help in bringing a better understanding of brain organization.

## Materials and methods

### Animals and tissue processing

For the present study embryos and larvae of *Xenopus laevis* were used. They were staged according to Nieuwkoop and Faber (1967) and sorted into embryonic (35–45), premetamorphic (46–52), prometamorphic (53–59), and metamorphic (60–65) stages (see Table 1). All animals were treated according to the regulations and laws of the European Union (2010/63/EU) and Spain (Royal Decree 53/2013) for care and handling of animals in research, after approval from the University Complutense to conduct the experiments described. Adult *Xenopus* were purchased from commercial suppliers (XenopusOne, Dexter MI), and the different developing specimens were obtained by breeding induced by chorionic gonadotropin (Pregnyl; Organon, West Orange, NJ) and kept in tap water at 20–25°C.

**Table 1:**
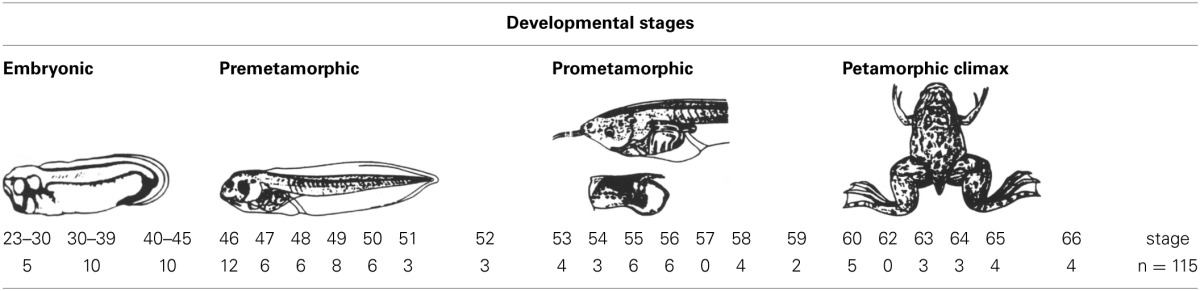
**Number of animals investigated at different stages of development for Pax7 immunohistochemistry**.

Staging of the embryos and larvae according to (Nieuwkoop and Faber, 1967).

At appropriate times, the animals were deeply anesthetized by immersion in a 0.4 mg/ml solution of tricaine methanesulfonate (MS222, Sigma Chemical Co., St Louis, MO) and perfused transcardially with 0.9% NaCl, followed by the fixative MEMFA (0.1 M MOPS [4-morpholinepropanesulphonic acid], 2 mM EGTA [ethylene glycol tetraacetic acid], 1 mM MgSO4, 3.7% formaldehyde) or 4% paraformaldehyde in 0.1 M phosphate buffer (PB, pH 7.4). The brains were dissected out and postfixed approximately 3–4 h in the same fixative solution at 4°C. At early developmental stages, when perfusion was technically impossible (between stages 26 and 47), the whole animal was fixed by immersion and processed. After fixation, the brains were immersed in a solution of 30% sucrose in PB until they sank. For sectioning on a freezing microtome (Thermo Scientific Microm HM 450) the tissue was embedded in a solution of 20% gelatin with 30% sucrose in PB, and stored overnight in a 10% formaldehyde solution with 30% sucrose in PB at 4°C. Brains were then sectioned at 15–30 μm in the transverse, sagittal or horizontal plane. Free-floating sections were collected and rinsed in PB.

### Immunohistochemistry

Immunohistofluorescence procedures were conducted for different primary antibodies, all of which were diluted in 5–10% normal goat serum in PB with 0.1% Triton X-100 (Sigma) and 2% bovine serum albumin (BSA, Sigma). Different protocols were carried out on free-floating sections or in toto (early embryos), with incubation in the primary antibodies for 72 h at 4°C. The dilution of each primary antibody used is detailed in Table 2.

**Table 2:** **Antibodies used in the present study**.

**Antigen**	**Immunogen**	**Type of antibody and commercial supplier**	**MW (kDa)**	**Dilution**
PAX7	*E. coli*-derived recombinant chick PAX7. Aa 352–523 of chick Pax7	Monoclonal mouse-anti-Pax7. Developmental studies hybridoma bank; catalog reference: PAX7	55	1:500
CR	Recombinant human CR	Polyclonal rabbit anti-CR. Swant, Bellinzona, Switzerland; catalog reference: 7699/4	29	1:1000
GABA	GABA-BSA	Polyclonal rabbit anti-c- minobutyric acid. Sigma, St. Louis, MO, USA; catalog reference: A2052	0.0103	1:3000
Otp	Amino acid sequence: RKALEHTVSMSFT of the C-tenninal OTP	Polyclonal rabbit-anti-Otp. Pikcell Laboratories, Kruislaan, Amsterdam, The Netherlands	34	1:1000
TH	Catalytic core of TH molecule protein purified from rat pheochromocytoma	Polyclonal rabbit anti-TH. Millipore (Chemicon); catalog reference: AB 152	62	1:1000
Nkx2.1	Amino acids 110–122 from the amino terminus	Polyclonal rabbit -anti-TTF. Biopat hnmunotechnologies, Caserta, Italy; catalog reference: PA 0100	42-37	1:500
NPY	Neuropeptide Y (porcine) conjugated to BS A	Polyclonal rabbit anti-NPY. Diasorin, Stillwater, MN, USA; catalog reference: N 22940	4.27	1:1000

Single-staining protocols for the detection of Pax7 were carried out on the free-floating sections as follows: (1) incubation for 72 h at 4°C in the dilution of the primary serum (see Table 2) in PB with 0.1% Triton X-100. (2) The second incubations were conducted with Alexa 488-conjugated goat anti-mouse (green fluorescence; Molecular Probes; Eugene, OR; catalog reference: A21042), diluted 1:500 for 90 min at room temperature: For bright field immunohistochemistry, free-floating sections were rinsed twice in PB, treated with 1% H_2_O_2_ in PB for 20 min to reduce endogenous peroxidase activity, rinsed again three times in PB, incubated in the primary antibody dilution (mouse anti-Pax7) with 0.025% Triton X-100 in PB, followed by incubation in biotinylated horse anti-mouse (1:100; Vector, Burlingame, CA; catalog reference: BA-2000), rinsed three times in PB, and visualized by the ABC-DAB kit method (Vector, SK4100).

To study the relative distribution of two markers in the same sections, the two-step protocol for immunohistofluorescence was used, with cocktails of pairs of primary antibodies (always developed in different species), at the same dilutions and conditions specified in Table 2. According to the species in which the primary antibody was raised, the second incubations were conducted with the appropriate fluorescent-labeled secondary antibody cocktails diluted in PB for 90 min at room temperature: Alexa 594-conjugated goat anti-rabbit (1:500), Alexa 488-conjugated goat anti-mouse (1:500), Alexa 594-conjugated donkey anti-goat (1:500; Molecular Probes; catalog reference: A11058), Alexa 594-conjugated chicken anti-rabbit (1:500; Molecular Probes; catalog reference: A21442), and fluorescein-conjugated rabbit anti-sheep (1:500; Vector; catalog reference: FI-6000). The sections were routinely counterstained with the nuclear marker Höechst (Sigma; Höechst 33258) to facilitate interpretation of the results. In all cases, after being rinsed the sections were mounted on glass slides and coverslipped with Vectashield mounting medium (Vector; catalog number: H1000).

### Controls and specificity of the antibodies

General controls for the immunohistochemical reaction included: (1) western blot analysis; (2) staining some selected sections with preimmune mouse, rabbit, or goat serum instead of the primary antibody; (3) controls in which either the primary and/or the secondary antibody was omitted. In all these negative controls the immunostaining was eliminated. In addition, all the antibodies used have been tested, under identical conditions, in tissues devoid of antigen (rat brain slices at levels revealing no expression), as negative control, and in tissues positive for the antigen (rat brain slices at levels expressing the antigen). In all cases the controls were satisfactory.

The specificity of the antibodies used has been assessed by the commercial companies (Table 2) and, in addition, the immunoblotting conducted in our previous studies with *Xenopus laevis* showed that all antibodies used labeled a single band, which corresponded well (with minor variations) to the bands labeled in the rat lanes (see Morona and González, 2008, 2009; Morona et al., 2011; Moreno et al., 2012a; Domínguez et al., 2013a).

### Evaluation and presentation of the results

**Figure 1:**
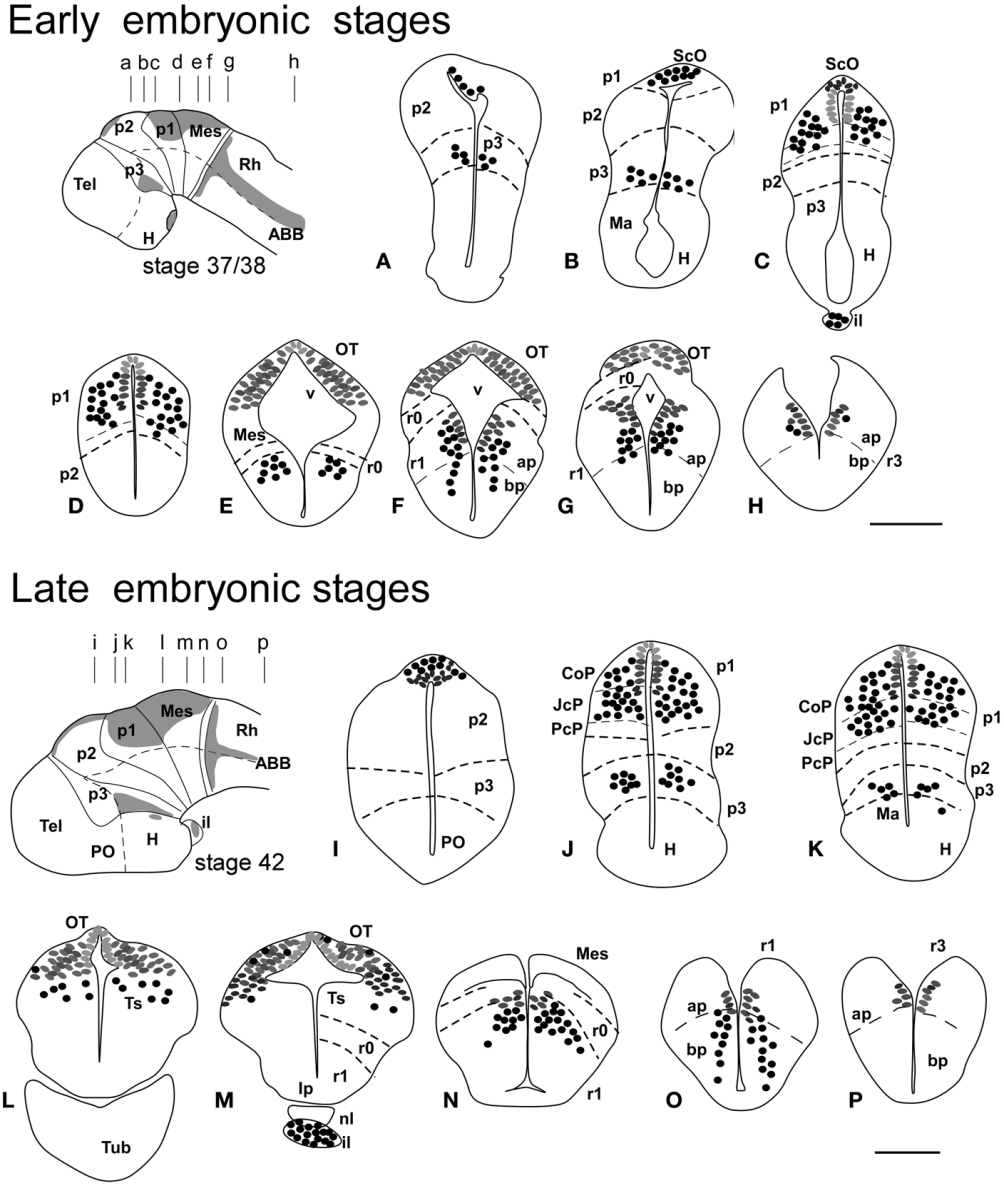
**Schematic drawings of transverse sections through the brain of *Xenopus laevis* at early embryonic stage 37/38 (A–H) and late embryonic stage 42 (I–P) showing the distribution of Pax7 immunoreactive cells**. The levels of the sections are indicated in the schemes of lateral views of the brain where the shaded areas represent the regions expressing Pax7. Scale bars = 100 μm. See list for abbreviations.

**Figure 2:**
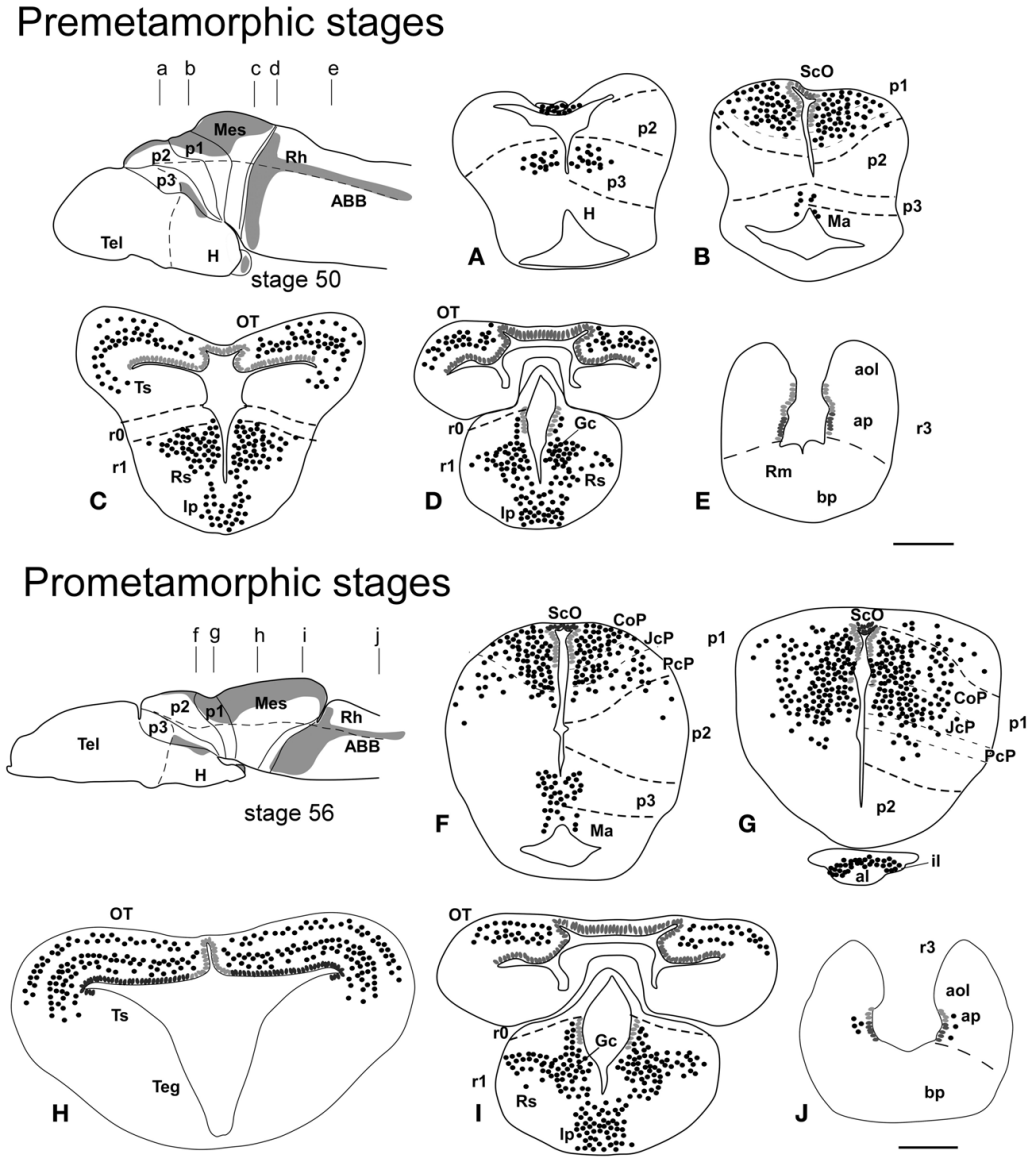
**Schematic drawings of transverse sections through the brain of *Xenopus laevis* at premetamorphic stage 50 (A–E) and prometamorphic stage 56 (F–J) showing the distribution of Pax7 immunoreactive cells**. The levels of the sections are indicated in the schemes of lateral views of the brain where the shaded areas represent the regions expressing Pax7. Scale bars = 200 μm. See list for abbreviations.

**Figure 3:**
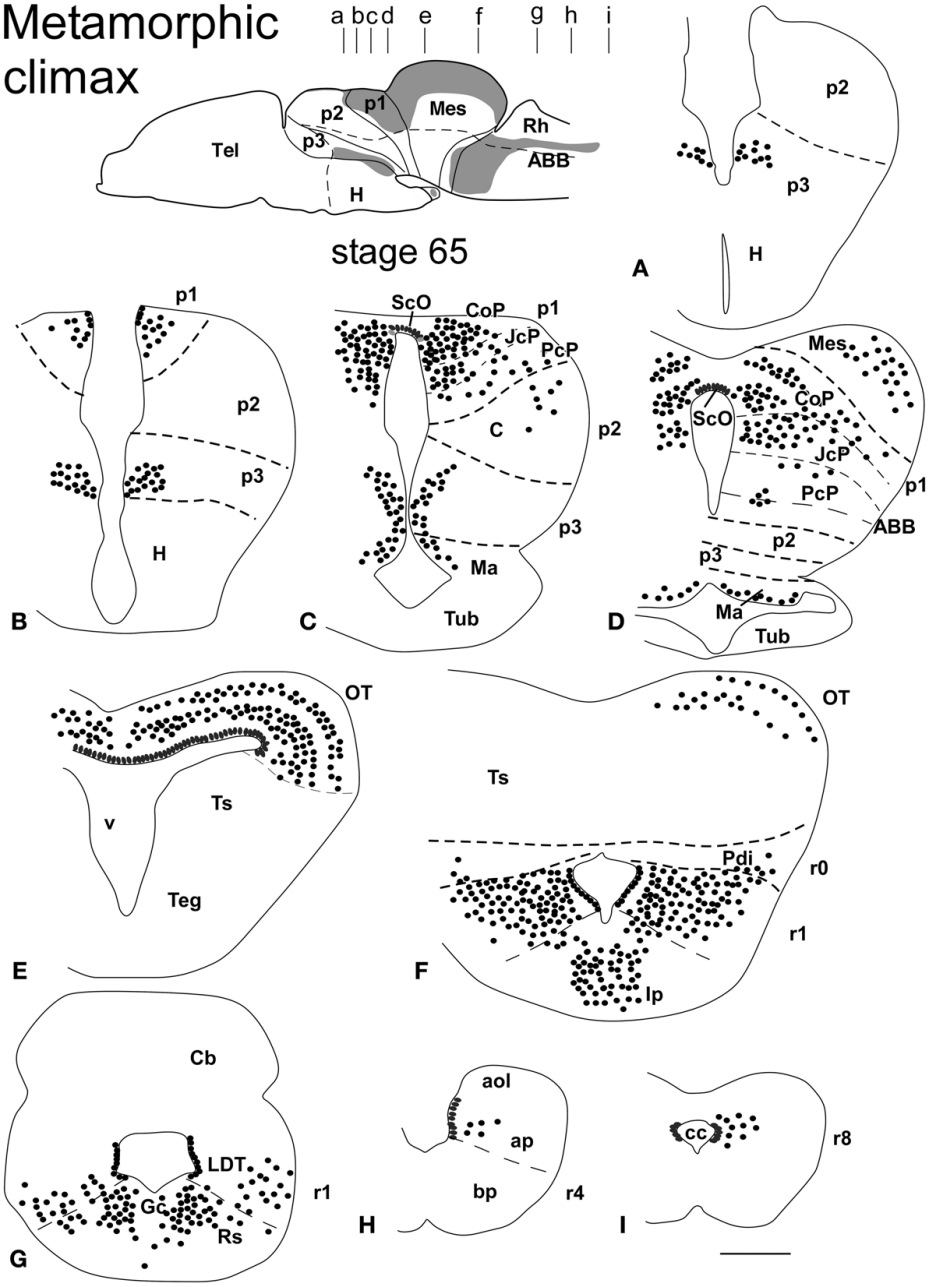
**Schematic drawings of transverse sections through the brain of *Xenopus laevis* at the metamorphic climax stage 65 (A–I) showing the distribution of Pax7 immunoreactive cells**. The levels of the sections are indicated in the scheme of the lateral view of the brain where the shaded areas represent the regions expressing Pax7. Scale bars = 400 μm. See list for abbreviations.

**Figure 4:**
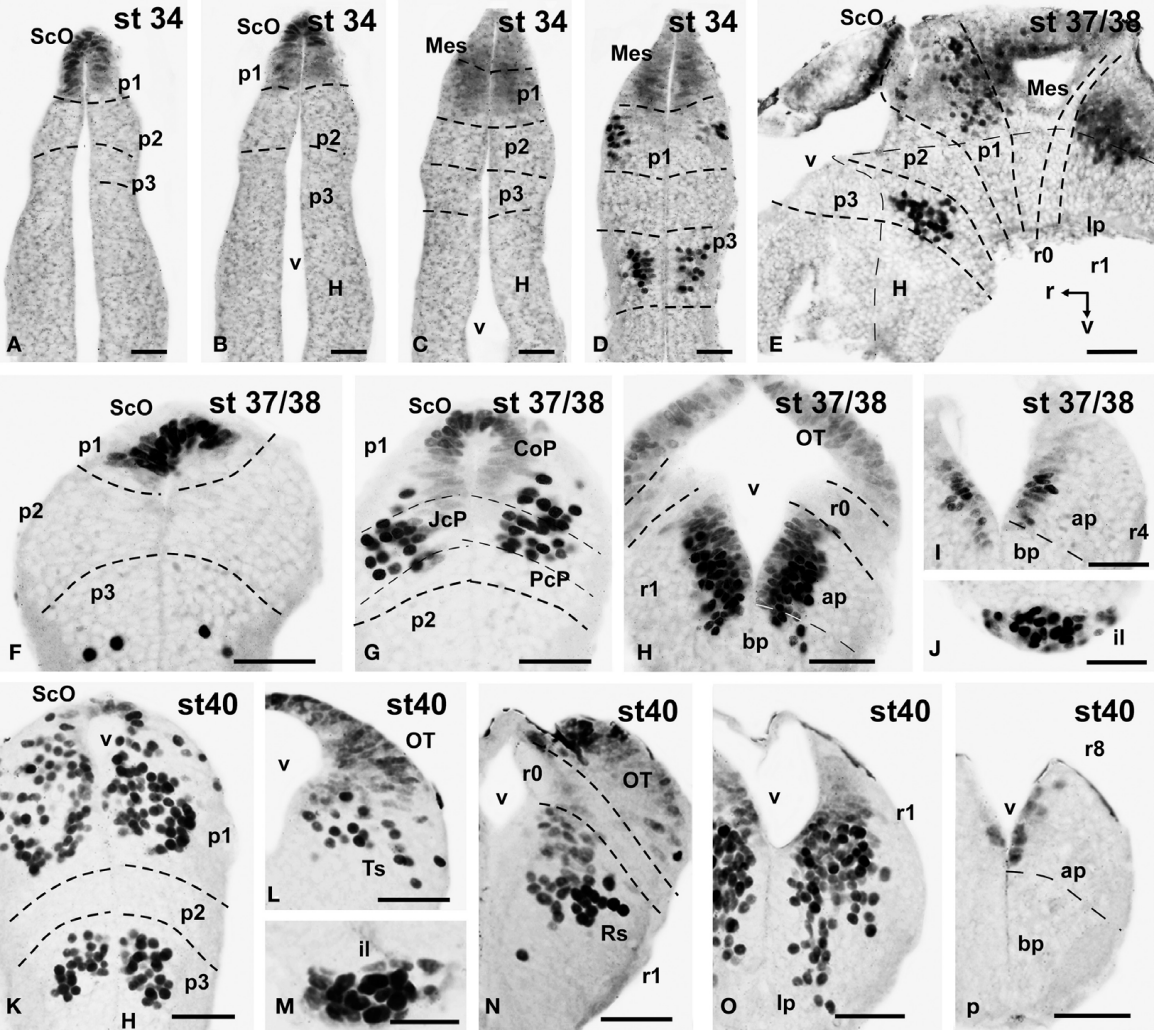
**Photomicrographs of single-stained transverse (A–D, F–P) and sagittal (E) sections through the brain of *Xenopus laevis* showing the localization of Pax7 cells**. Different stages (indicated in the upper right corner of each photomicrograph) corresponding to early embryos are illustrated. All images are oriented following the same standard: dorsal is upwards in transverse and sagittal sections, and rostral is to the left in sagittal sections. The neuromeric boundaries and main brain subdivisions are indicated to assist in the precise localization of the labeled cells. Scale bars = 50 μm. See list for abbreviations.

**Figure 5:**
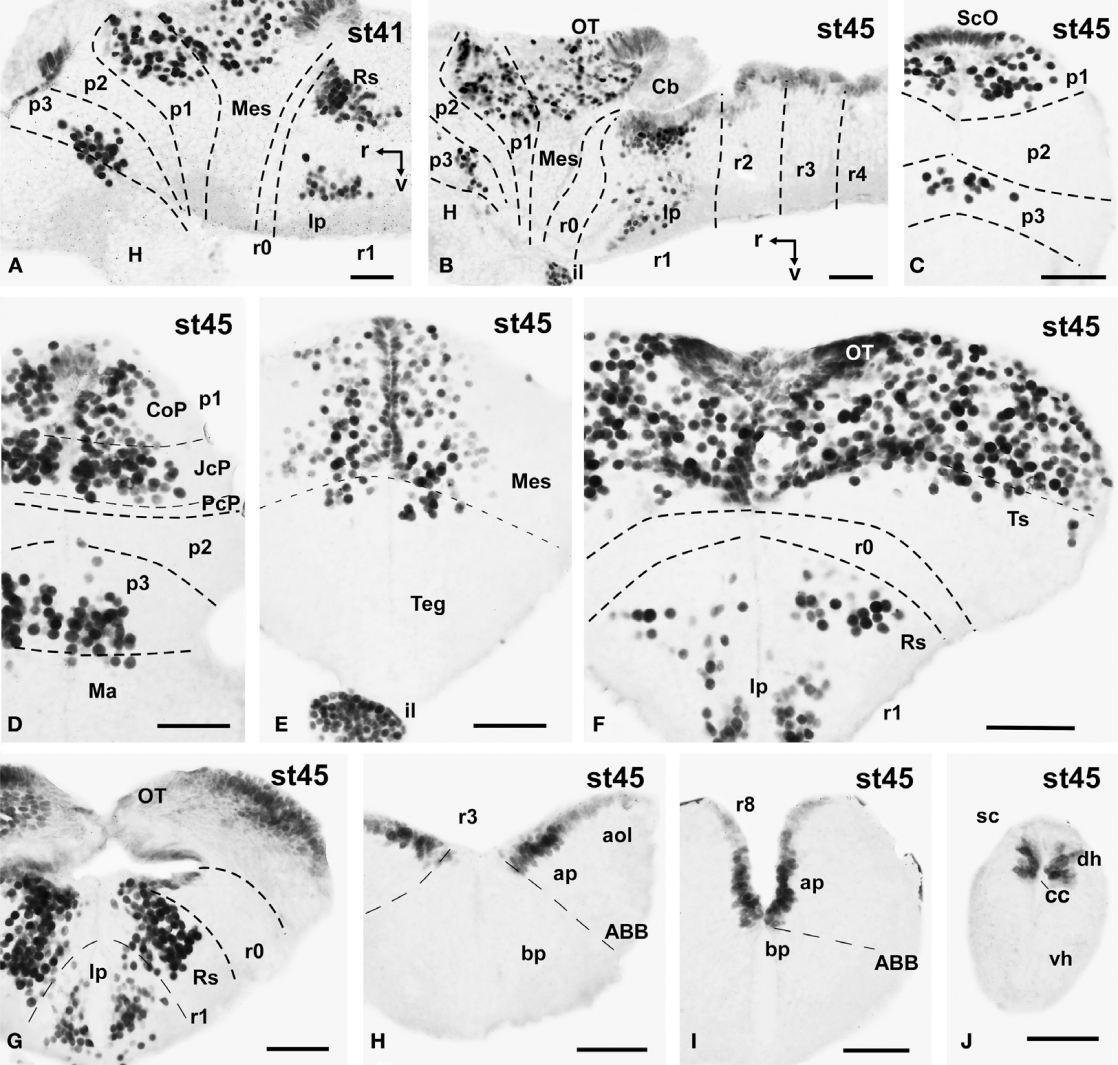
**Photomicrographs of single-stained sagittal (A,B) and transverse (C–J) sections through the brain of *Xenopus laevis* showing the localization of Pax7 cells**. Different stages (indicated in the upper right corner of each photomicrograph) corresponding to late embryos are illustrated. All images are oriented following the same standard: dorsal is upwards in transverse and sagittal sections, and rostral is to the left in sagittal sections. The neuromeric boundaries and main brain subdivisions are indicated to assist in the precise localization of the labeled cells. Scale bars = 50 μm. See list for abbreviations.

**Figure 6:**
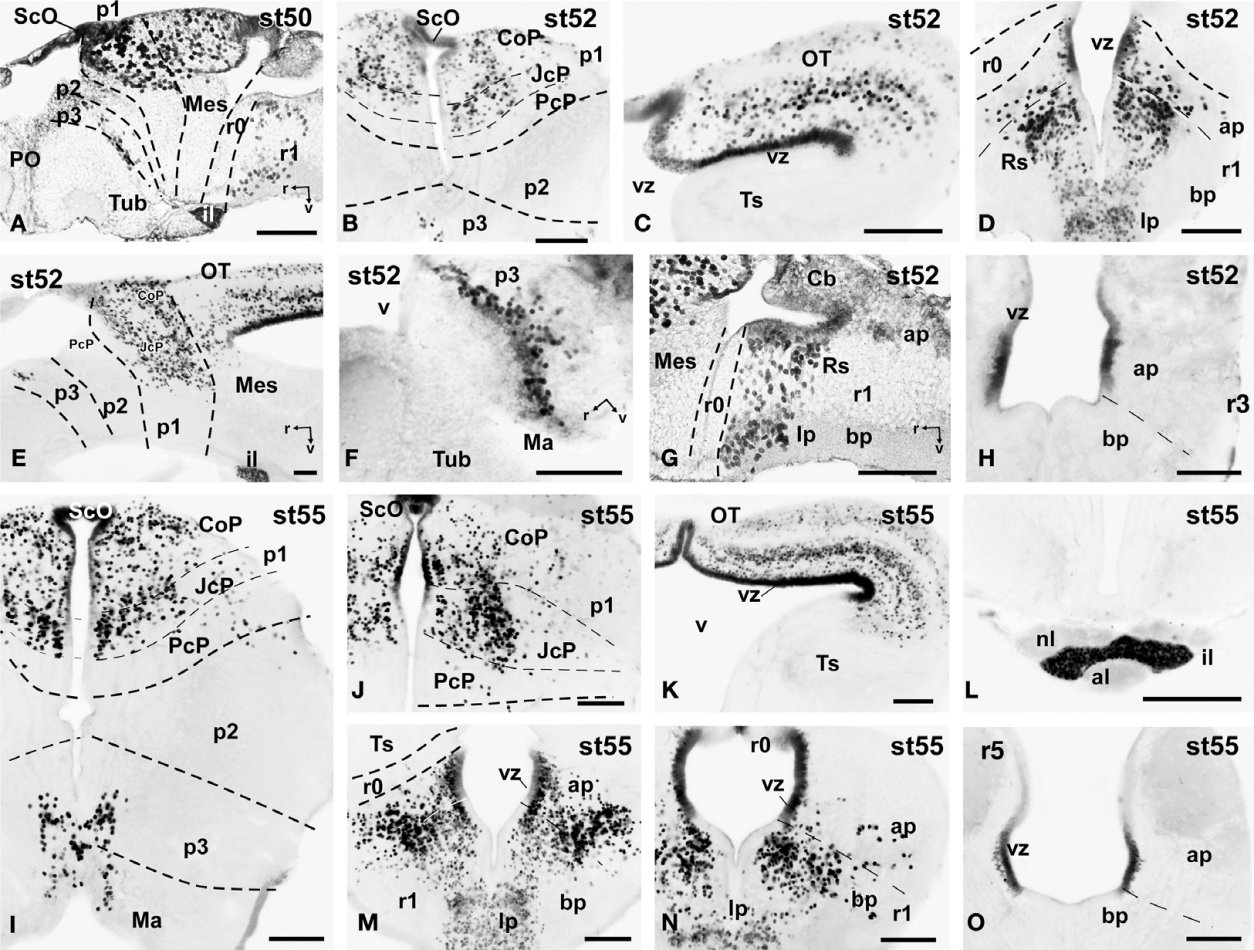
**Photomicrographs of single-stained sagittal (A,E-G) and transverse (B–D, H-O) sections through the brain of *Xenopus laevis* showing the localization of Pax7 cells**. Different stages (indicated in the upper right corner of each photomicrograph) corresponding to premetamorphic **(A–H)** and prometamorphic **(I–O)** tadpoles are illustrated. All images are oriented following the same standard: dorsal is upwards in transverse and sagittal sections, and rostral is to the left in sagittal sections. The neuromeric boundaries and main brain subdivisions are indicated to assist in the precise localization of the labeled cells. Scale bars = 100 μm. See list for abbreviations.

**Figure 7:**
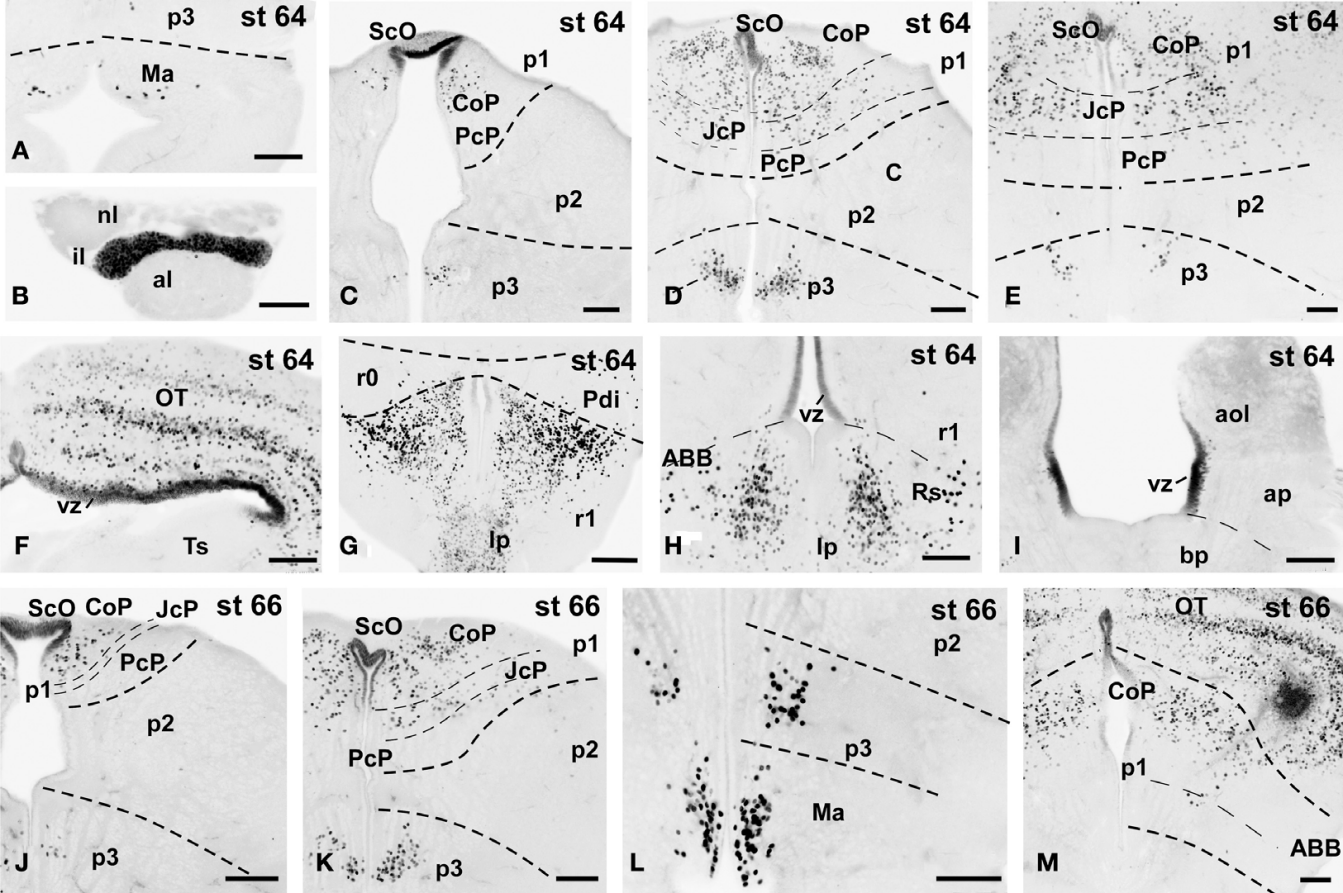
**Photomicrographs of single-stained transverse (A–M) sections through the brain of *Xenopus laevis* showing the localization of Pax7 cells**. Different stages (indicated in the upper right corner of each photomicrograph) corresponding to tadpoles at the climax of the metamorphosis are illustrated. In all transverse images dorsal is upwards. The neuromeric boundaries and main brain subdivisions are indicated to assist in the precise localization of the labeled cells. Scale bars =100 μm. See list for abbreviations.

**Figure 8:**
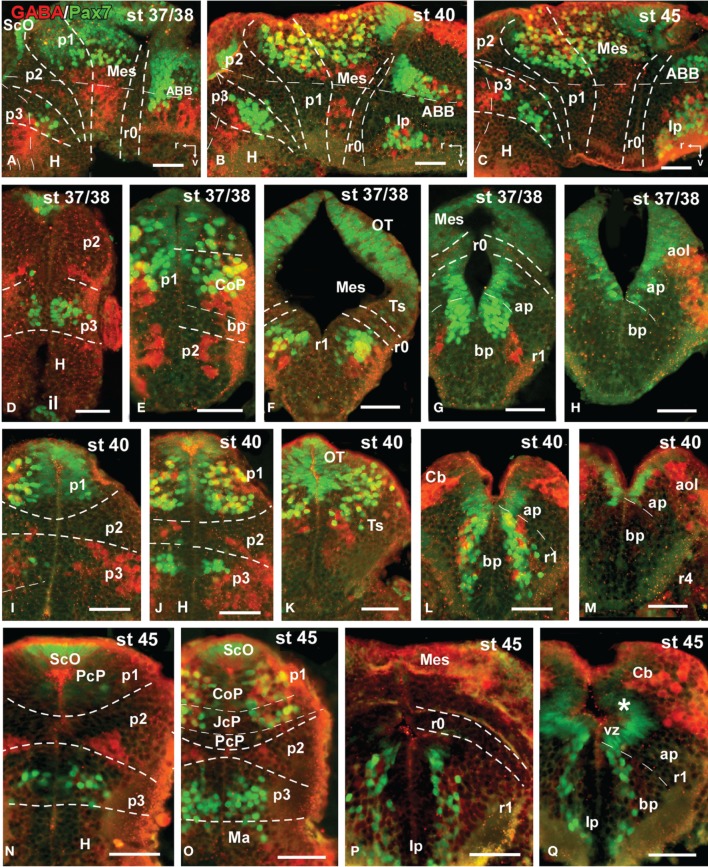
**Photomicrographs of double-labeled sagittal (A–C) or transverse (D–Q) sections through the brain of *Xenopus laevis* showing the localization of Pax7 cells (gree fluorescence) in relationship to GABA (red fluorescence) at different embryonic stages (indicated in the upper right corner of each photomicrograph)**. All images are oriented following the same standard: dorsal is upwards in transverse and sagittal sections, and rostral is to the left in sagittal sections. The neuromeric boundaries and main brain subdivisions are indicated to assist in the precise localization of the labeled cells. Asterisk in **(Q)** indicates the weak expression found in the ventricular zone of the developing cerebellum. Scale bars = 50 μm. See list for abbreviations.

**Figure 9:**
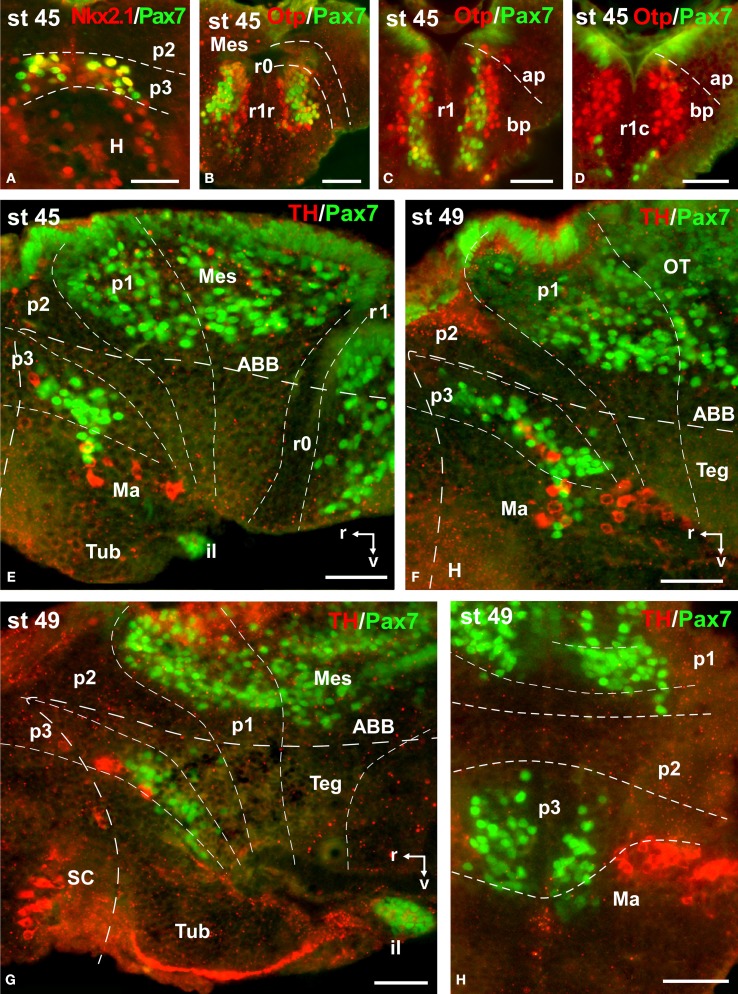
**Photomicrographs of double-labeled transverse (A–D,H) and sagittal (E–G) sections through the brain of *Xenopus laevis* showing the localization of Pax7 cells (green fluorescence) in relation to the markers Nkx2.1, Otp, and TH (red fluorescence; indicated in color code in the upper right corner of each photomicrograph)**. The developmental stages are indicated in the upper left corner of each photomicrograph. All images are oriented following the same standard: dorsal is upwards in transverse and sagittal sections, and rostral is to the left in sagittal sections. The neuromeric boundaries and main brain subdivisions are indicated to assist in the precise localization of the labeled cells. Scale bars = 50 μm. See list for abbreviations.

**Figure 10:**
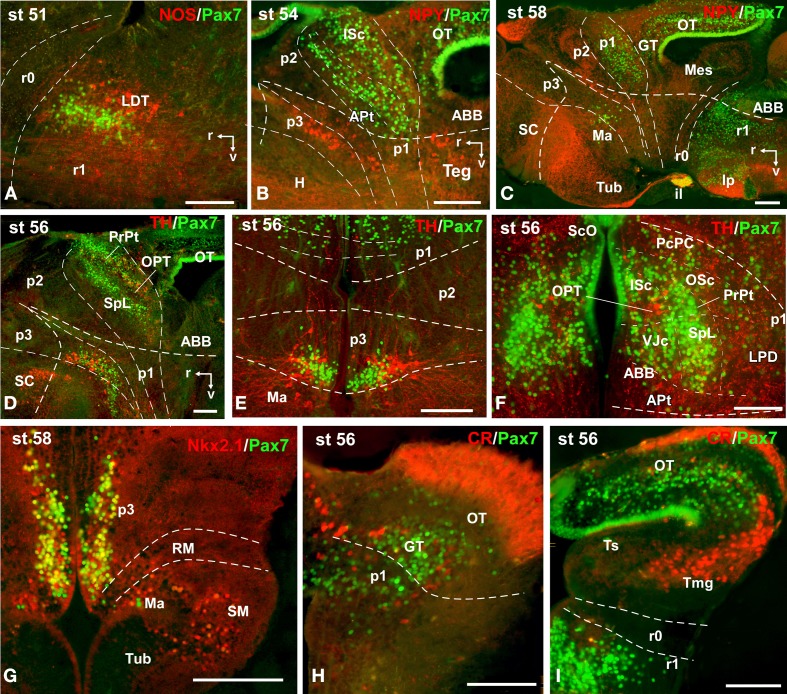
**Photomicrographs of double-labeled sagittal (A–D) and transverse (E–I) sections through the brain of *Xenopus laevis* showing the localization of Pax7 cells (green fluorescence) in relation to the markers NPY, TH, CR, Nkx2.1, and NOS (red fluorescence; indicated in color code in the upper right corner of each photomicrograph)**. The developmental stages are indicated in the upper left corner of each photomicrograph. All images are oriented following the same standard: dorsal is upwards in transverse and sagittal sections, and rostral is to the left in sagittal sections. The neuromeric boundaries and main brain subdivisions are indicated to assist in the precise localization of the labeled cells. Scale bars = 100 μm. See list for abbreviations.

The distribution of Pax7 cells in the brain of *Xenopus* was charted in selected transverse sections at representative developmental stages (Figures 1–3). Their relative localization was framed attending to the neuromeric organization of the brain. We used the same nomenclature as in our previous studies (Morona and González, 2008, 2009, 2013; Domínguez et al., 2011, 2013a,b; Morona et al., 2011). Single and double-labeled sections were analyzed with an Olympus BX51 microscope equipped for fluorescence with appropriate filter combinations, and selected sections were photographed using a digital camera Olympus DP72. Photomicrographs were adjusted for contrast and brightness with Adobe PhotoShop CS4 (Adobe Systems, San Jose, CA) and were mounted on plates (Figures 4–10) using Canvas 11 (ACS Systems International, Santa Clara, CA). The schematic drawings were also made with the Canvas 11 software.

## Results

The distribution patterns of Pax7 immunoreactive cells were analyzed in the CNS throughout the embryonic and larval development of *Xenopus laevis*. For each stage the pattern of distribution and intensity of the immunoreaction were consistent among animals treated identically. One advantage of this species for developmental studies is that the ontogeny extends over a rather long period of time (58–60 days) and that the development of external features makes it easy to characterize distinct stages (Nieuwkoop and Faber, 1967). The long larval period, which starts with independent feeding, is generally subdivided into three stages (see González et al., 1994; Morona and González, 2013): (1) premetamorphic stages, in which the tadpole merely grows in size and the buds of the hindlimbs appear on the lateral side of the body; (2) prometamorphic stages, characterized by progressive formation of the hindlimbs; and (3) metamorphic climax, marking the period in which the transformation of the tailed larval form into the tailless, four-legged juvenile occurs. The brain of recently metamorphosed juveniles (stage 65–66) already shows all main anatomical characteristics as in the adult brain. In the following sections, we describe the spatiotemporal sequence of appearance of Pax7 cells for these periods of development. To help the flow of the description, a timetable of the appearance of Pax7 cell groups in the developing brain of *X. laevis* is shown in Table 3.

**Table 3:**
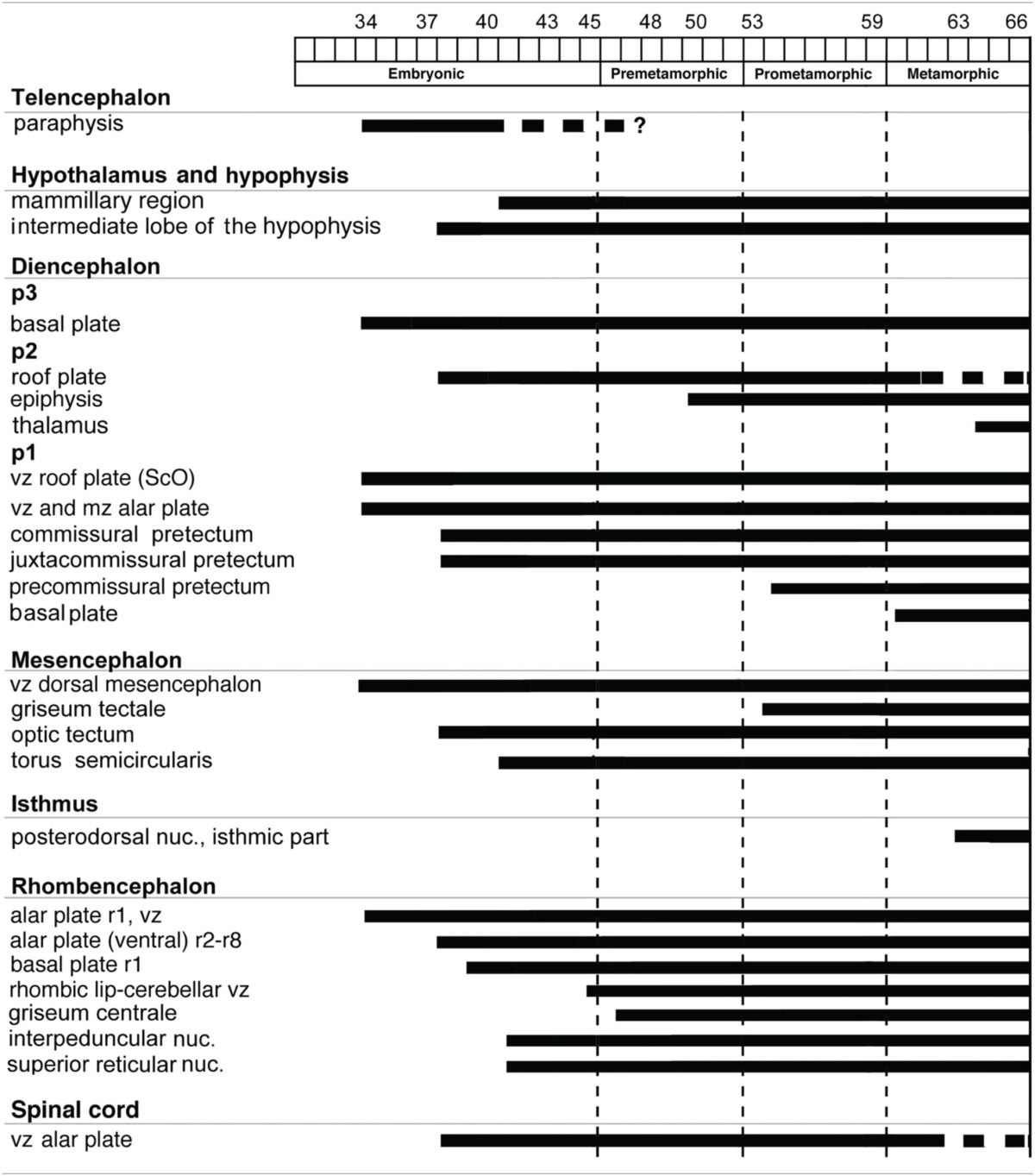
**Timetable of appearance of Pax7 cell groups in the CNS of *Xenopus laevis***.

For the description of the results, we will consider the main subdivisions of the brain of *Xenopus laevis* that can be recognized at the particular developmental stages (Morona and González, 2013). Pallial and subpallial regions will be considered in the telencephalon, and alar (supraoptoparaventricular and suprachiasmatic) and basal (tuberal and mammillary) regions in the hypothalamus. Topologically caudal, the diencephalon is subdivided into three segments, prosomeres 1–3 (p1–p3). These contain in their alar regions the prethalamus plus the prethalamic eminence in the rostral p3, the thalamus plus the habenula or epithalamus in the intermediate p2, and the pretectum in the caudal p1. The smaller basal components form the tegmental region in the diencephalon, extending in the three prosomeres (Puelles and Rubenstein, 2003; reviewed in Puelles et al., 2012b). The mesencephalon is considered a single segment containing the optic tectum and the torus semicircularis dorsally and the midbrain tegmentum ventrally. The isthmus is called r0 and rhombomeres 1–8 are indicated in relation to the motor nuclei of the cranial nerves (López et al., 2002; Morona and González, 2013). In addition, we will refer to expression in the ventricular zone (vz; cells that are in close contact with the ventricle), the adjacent subventricular zone (svz; one-to-two rows of tightly packed cells), and the mantle zone (mz; migrated cells in the external rows of the cell layer and into the superficial fiber zone). In general, we will describe the results at each developmental stage from rostral to caudal brain regions. The relevant data on the codistribution between Pax7 with the other markers used will be commented upon for each developmental period, along with a description that will help in the understanding of the precise localization and signature of many cell groups.

### Early embryonic stages (34–40)

The first Pax7 cells were detected by stage 34 (about 44 h postfertilization) just after the animals exhibit the first spontaneous movements. The cells located most rostrally were found in the basal plate of the rostral portion of the diencephalic vesicle, in the prospective p3 (Figure 4D). Caudally, lightly stained cells occupied the vz of the alar part of p1 (Figures 4A–C), whereas intensely labeled cells were present in the mz (Figure 4D) and in the subcommissural organ (ScO), a roof plate derivative in p1 (Figures 4A,B). In addition, weak staining was detected in dorsal mesencephalic vesicle (Figures 4C,D) and in the alar plate of the rostral hindbrain. Of note, in these early stages that were processed in toto, weak immunoreactivity was observed in the membranous structures between the telencephalic hemispheres that contain the paraphysis, distinct from the choroid plexus. However, the continuity of this expression in subsequent developmental stages could not be assessed in our material (see Discussion).

By stages 37/38 (about 60 h postfertilization), the intensity and the number of Pax7 positive cells increased, especially in the mz (Figures 1A–H). Strongly Pax7 expressing cells occupied mainly the dorsal part of the basal plate in p3 (Figures 1A,B, 4E, 8A,D) and Pax7 expression was detected for the first time in the roof plate of p2 (Figures 1A, 8A,D). Pax7 labeled cells were also detected in the developing hypophysis representing the intermediate lobe identifiable at later stages (Figures 1C, 4J, 8D). Within p1, the intensity of the Pax7 expression markedly increased in the ScO (Figures 1B,C, 4E,F, 8A) and the mz of the PT (Figures 1C,D, 4E,G, 8E), whereas weak staining was observed in the vz only in the caudal PT (Figure 4G). The combination with GABA highlighted the lack of Pax7 cells in the developing precommissural part of the pretectum (PcP) in contrast to the juxtacommissural (JcP) and commissural (CoP) parts (Figure 8E). Weak staining continued caudally along the mesencephalon in the developing optic tectum, in which practically all cells seemed to express Pax7 (Figures 1E–G, 4H, 8F,G). The isthmus, which is curved and thin at these stages and consisting in a few rows of cells, was characterized by the absence of Pax7 and GABA staining (Figures 1E–G, 4E,H, 8A,F,G). In the rostral part of r1, Pax7 cells were labeled in the ventricular layer (Figures 1F,G, 8G) and the cells were more intensely stained as they get displaced from the ventricle and detach ventrally (Figures 4H, 8G). The ventrally located cells most likely were located in the basal plate, although this was better observed in subsequent stages. Some of the Pax7 cells observed in the rostral part of r1 showed also immunoreactivity for GABA (Figure 8F), whereas caudally they formed separate populations (Figure 8G). Caudally in r1, the vz and svz of the alar plate expressed Pax7 with higher intensity in its ventral part and this expression continued caudally along the rhombencephalon (Figures 1H, 4I, 8H) and the rostral spinal cord.

The end of the early embryonic period is considered when the mouth of the embryo breaks through (stage 40; 2 days and 18 h). The brain at this stage showed higher numbers of Pax7 cells than in previous stages. The Pax7 cell population in the basal part of p3 was strongly stained, and some cells might extend into basal hypothalamic territories (Figure 4), as observed in latter stages. Conspicuous Pax7 expression was also observed in the hypophysis (Figure 4M). Pax7 expression continued in the roof plate of p2 and p1 (Figure 4K). In the pretectal region, only weakly Pax7 expressing cells were observed in the vz (Figures 4K, 8J), whereas more intensely labeled Pax7 cells were observed in the svz and external mz, and numerous cells in the mantle zone were double labeled for Pax7 and GABA (Figures 8B,I,J). In the mesencephalon, the dorsal part of the tectum contained a large population of Pax7 cells, whereas ventrolaterally only some scattered cells were found primarily in its caudal part, and labeled cells were also found in the torus semicircularis (Figures 4L, 8K). The isthmus (r0) was highlighted by the lack of Pax7 cells, in contrast to the abundant populations in the adjacent mesencephalon and r1 (Figure 8B). Along the rhombencephalon and the upper spinal cord Pax7 was expressed in the vz of the alar plate, more intensely in the ventral domain than in the dorsal domain where expressing cells were not frequently observable caudal to r4 (the level of the facial motor nucleus) (Figures 4O,P, 8M). Strikingly, in the rostral part of r1 strongly Pax7 expressing cells were found separated from the ventricle and extending laterally and ventrally into the superior reticular region (later superior reticular nucleus) and into the location where the interpeduncular nucleus will develop (Figures 4N,O). These Pax7 cells were intermingled with some GABAergic cells, but actual colocalization in the same cells was not observed (Figure 8L).

### Late embryonic stages (41–45)

The late embryonic stages begin when the tadpoles swim spontaneously, and extend for less than 3 days absorbing the remaining yolk, ending with the independent feeding of the larval tadpole. During the five stages of this period the brain changes considerably and most Pax7 cell populations grow in size and complexity (Figures 1I–P).

During this period, the cells located in the basal part of p3 were seen progressively more ventrally (topologically rostral) in the hypothalamus (compare Figures 1K, 5A,B,D, 8O), and these Pax7 cells extended medially in a column close the infundibular ventricle, close to the TH positive cells of the mammillary region but forming separate populations (Figure 9E). Some of these Pax7 cells at the limit between p3 and the mammillary region showed immunoreactivity for Nkx2.1 (Figure 9A) and they were medially located to the abundant GABA positive cell population of p3, as observed already from stage 40 (Figure 8J). Also at these stages, cells were intensely Pax7 immunoreactive in the intermediate lobe of the hypophysis (Figures 1M, 5B,E). Pax7 cells in p2 were only present along the roof plate (Figures 1I, 9E), whereas they were strikingly abundant in p1 within the PT where they progressively segregated into distinct pretectal territories. Thus, in addition to the ScO in the roof plate, distinct Pax7 cells occupied the intermediate (JcP) and the caudal (CoP) domains of the pretectum (Figures 1J,K, 5D, 8O). Of note, Pax7 was expressed in the vz and mz of CoP, whereas the vz of the JcP was virtually devoid of expressing cells. In turn, the PcP domain was characterized at these stages by the absence of Pax7 cells (Figures 1J,K, 5D, 8O).

Caudal to the diencephalo-mesencephalic boundary Pax7 cells were present in the vz throughout the optic tectum (Figures 1l,M, 5E,F). We also observed a large number of migrated cells in the optic tectum, where they populated all developing cell layers (Figures 1L,M, 5E,F). Within the dorsal mesencephalon, the torus semicircularis also showed some migrated Pax7 cells, but they were absent in the ventricular lining (Figures 1L,M, 5F, 8K), as in previous stages. The isthmic segment (r0) was devoid of Pax7 cells (Figures 1M,N, 5F,G, 8P). The cerebellar anlage developed laterally in the dorsal r1 containing abundant GABA positive cells and the moderate Pax7 staining in the vz of the most dorsal alar region probably represents the rostral rhombic lip (asterisk in Figure 8Q). From r1 to the spinal cord, Pax7 was continuously expressed in the vz of the alar plate, as described in the early embryonic stages (Figures 1O,P, 5H,J, 8Q). The Pax7 cells found ventrally at the r0-r1 boundary were located lateral to the Otp positive cells, whereas more caudally the Pax7 cells were located medial to the Otp cell group (Figures 9B–D), and some of these cells that occupied the basal plate were double labeled in the rostral portion of r1 (Figures 9B, C). The population of cells situated a long distance from the ventricle occupied the superior reticular zone (laterally) and the interpeduncular nucleus (ventromedially) (Figures 5B,F,G, 8P,Q). In the rhombencephalic alar plate, the Pax7 cells in the ventricular zone were intensely labeled just medial to the developing nuclei of the octavolateral area, which contained GABAergic cells, between r1-r4 (Figure 8M). In the caudal rhombencephalon and spinal cord, the ventricular staining pattern in the alar vz was similar to that described for previous stages (Figure 5J).

### Premetamorphic stages (46–52)

During the first period of the larval life, the premetamorphic larvae develop active feeding and free-swimming while the hindlimb buds develop on the laterals of the body. At these stages the main brain structures and subdivisions were recognized and cell populations became gradually more segregated. The intensity of the immunoreaction increased in most cell groups, and new Pax7 cell populations were distinctly identified. The most topologically rostral Pax7 cells were observed in the hypothalamic mammillary region (Figures 2B, 6F). These few cells were identified in the hypothalamus in sections double labeled for TH/Pax7 (Figures 9F–H). The intensely labeled Pax7 cells of the intermediate lobe of the hypophysis increased in number along this period (Figures 6A,E, 9G).

As in previous stages, the most striking labeling in the diencephalon was noted in a cell band in the basal p3 (Figures 2A, 6A,F, 9F–H). Intensely positive Pax7 cells were also labeled in the roof plate of p2, likely in the epiphysis, and along the roof ventricular zone and in the subcommissural organ in p1 (Figures 2A,B, 6A,B). The Pax7 expression pattern observed in the pretectum in earlier stages was largely maintained although the number of labeled cells increased (Figures 2B, 6A,B,E, 9H).

The mesencephalon at these stages showed a numerous Pax7 cell population distributed in all developing cell layers of the optic tectum, especially in the vz and in the intermediate (intensely labeled cells) and superficial (lightly labeled cells) layers. Only a few Pax7 cells were detected in the torus semicircularis, where no expression was observed in the vz (Figures 2C,D, 6C,E).

In the rhombencephalon, the vz of the alar plate, mainly its ventral part, was positive for Pax7 as a continuation of the ventricular expression found in the mesencephalon, with only a small gap in r0 (Figures 2C–E, 6D,G,H). The cell groups in r1 were strongly reactive for Pax7, and the labeled cells progressively extended rostrally in the basal plate, segregating into different populations that were identified at these stages as the superior reticular nucleus, the griseum centrale, and the interpeduncular nucleus (Figures 2C,D, 6D,G). Of note, in the rostral r1 a particular group of densely packed Pax7 cells was observed in the alar plate medial and ventral to the laterodorsal tegmental nucleus (NOS immunoreactive; Figure 10A).

### Prometamorphic stages (53–59)

During prometamorphic stages (approximately 21 days) the development of the hindlimbs and fingers is accomplished. This period ends before the major macroscopic changes that transform the tadpole into a froglet take place. The brain along these stages increases markedly in size and all major subdivisions, anatomical landmarks and main cell populations could be distinguished as in the adult (Figures 2F–J).

A noticeable change observed during the prometamorphosis, as regards Pax7 cell distribution, was the progressive segregation of the hypothalamic mammillary population from the subgroup of the basal p3 (Figures 2F, 6I). At these stages, the labeled Pax7 cells located in the mammillary band did not express Nkx2.1, whereas those located in p3 coexpressed Nkx2.1 (Figure 10G). The Pax7 cell population of the basal p3 was arranged close to the ventricular lining and, more clearly than in previous stages, formed a separate population from the catecholaminergic cells located around them (Figures 9F–H, 10D,E). Pax7 immunoreactivity was very conspicuous in the hypophysis and in this period these labeled cells could be clearly identified solely in the intermediate lobe (Figures 2G, 6L), which is densely innervated by TH (not shown) and NPY (Figure 10C) fibers. Within the pretectal region, different nuclei could be distinguished by the distinct cell packing, the intensity of the Pax7 immunoreaction (Figures 6I,J), and by the relationship with other pretectal populations, such as the TH containing cells exclusively expressed in the homologous of the olivary pretectal nucleus (OPT), which was negative for Pax7 (Figures 10D,F). By these stages, the complex pretectal region showed a large Pax7 cell population clearly distributed in the different nuclei that mainly constitute the derivatives of the CoP and JcP regions of the previous stages (Figures 2F,G, 6I,J). Thus, Pax7 cells were observed in numerous pretectal nuclei previously identified in *Xenopus laevis* (see Morona et al., 2011), namely in the inner and outer nuclei of the ScO (ISc, OSc; Figures 10B,F), the spiriformis lateralis and principal pretectal nuclei (SpL, PrPt; Figures 10D,F), the parvocellular nucleus of the posterior commissure (PcPC; Figure 10F) and ventral subdivision of the juxtacommissural nucleus (VJc; Figure 10F). In addition, scattered cells were observed in the lateral posterodorsal and anterior pretectal nuclei (LPD, APt; Figures 10B,F). Of note, some Pax7 cells were now seen most laterally in the pretectal precommissural territories (Figure 6I) and scattered cells might even be located within the thalamus, in p2 (Figures 2G, 6I).

In the mesencephalon, the optic tectum strikingly grows during the prometamorphosis (compare Figures 2C,H) and abundant Pax7 cells were located in the griseum tectale (rostrally) and in the optic tectum, following the layered pattern observed in premetamorphic stages. The combination of CR immunoreactivity, which labels fibers in the whole marginal zone of the tectum and a distinct cell population from the Pax7 cells, allowed to observe a rostral decrease in the number of Pax7 cells with respect to the caudal part (Figures 10H,I). Only a few Pax7 cells could be seen within the dorsal part of the torus semicircularis but did not enter the magnocellular nucleus of the torus, highlighted by the CR staining (Figures 2H, 6K, 10I).

The Pax7 cell populations in the rostral rhombencephalon showed a more segregated pattern of distribution than in previous stages. They were clearly identified as cells in the proliferative vz and svz in the ventral part of the alar plate, as part of a migrated population along the alar and intermediate basal zones, and within the interpeduncular nucleus (Figures 2I, 6M,N). Along the rhombencephalon (Figure 6O) and in the spinal cord, in addition to the Pax7 cells of the ventral part of the alar vz detected in previous stages, some cells occupied also subventricular domains.

### Metamorphic climax (60–66)

The metamorphic climax is a short period (approximately 10–12 days), characterized by the transformation of the tadpole into a juvenile, with the resorption of the tail and change in locomotor behavior, now depending mainly on the limbs. Concerning Pax7 labeling, only a few differences were noted compared with earlier stages, but the segregation of the distinct Pax7 cell populations strikingly increased (Figures 3A–L, 7A–M). In general, Pax7 cells were better identified in the mammillary region of the hypothalamus (Figures 3C,D, 7A, L) and the conspicuous intermediate lobe of the hypophysis (Figure 7B). The Pax7 staining in the roof plate of p2 was virtually absent, whereas in the roof plate of p1 the distinct labeling of the ScO was maintained (Figures 3C,D, 7C-E,J,K). The large Pax7 cell population in the pretectal region followed a distribution pattern similar to that observed in previous stages, but more labeled cells were detected into the ventrocaudal PcP region and Pax7 isolated cells could be observed in the thalamus, lateral to the large central nucleus (Figures 3C,D, 7D,E,K). Also in the brain of metamorphosed *Xenopus* could a small population of Pax7 cells be observed for the first time in, or close to, the p1 basal plate (Figures 3D, 7M). Within the optic tectum, the ventricular expression was intense and the layered arrangement of labeled cells was maintained (Figures 3E,F, 7F,M). In the rhombencephalon, the numerous Pax7 cells extended rostrally into the isthmic segment in the posterodorsal tegmental nucleus (Pdi; Figures 3F, 7G). The Pax7 expression in the rostral rhombencephalon (r1) was maintained and the number of labeled cells increased in all cell populations described in previous stages, mainly in the superior reticular nucleus, the central gray and the interpeduncular nucleus (Figures 3F,G, 7G,H). In the caudal rhombencephalon and rostral spinal cord the number of labeled cells separated from the vz of the ventral alar plate increased (Figures 3H,I, 7I) and the ventricular expression in the spinal cord disappeared soon after the metamorphosis is completed.

## Discussion

We examined the Pax7 expression patterns during the ontogenetic development of the CNS of *Xenopus laevis*. The present immunohistochemical approach yielded high-resolution staining of cell nuclei from early stages. In similar studies in other species, the use of immunohistochemistry for the detection of the Pax proteins and *in situ* hybridization for the localization of Pax mRNA resulted in comparable patterns of staining (Hitchcock et al., 1996; Wullimann and Rink, 2001; Moreno et al., 2008a,b; González and Northcutt, 2009; Morona et al., 2011; Ferreiro-Galve et al., 2012; Lorente-Cánovas et al., 2012). The study of this transcription factor in combination with other markers, previously studied in *Xenopus* development (González et al., 1994; Moreno et al., 2008b; Morona and González, 2013), has been shown valuable for the interpretation of the precise localization of Pax7 positive cells, which is a difficult task particularly through ontogeny.

Notably, the expression patterns described herein throughout development were consistently interpreted within the current model of brain segmentation based on spatially restricted developmental gene expression patterns and their topological position within the neural tube, which is valid for all vertebrates (Gilland and Baker, 1993; Marín and Puelles, 1995; Puelles et al., 1996; Fritzsch, 1998; Cambronero and Puelles, 2000; Díaz et al., 2000; Puelles and Rubenstein, 2003; Straka et al., 2006). Pax7 is expressed from early development in specific regions that highly correlated with areas comprised in distinct segments, respecting the main boundaries between them. However, as development proceeds the progressive localization of Pax7 cells in some regions, as in the hypothalamus and basal rhombencephalon, suggests cell migration through development, as it was also proposed in the ontogeny of the urodele brain, where cell migrations are thought to be more highly restricted than in anurans (Joven et al., 2013b).

### Comparative Pax7 expression in *Xenopus* and other vertebrates

The comparison of our results in *Xenopus* with those previously described for representatives of other vertebrate groups highlights the conserved domains that express the Pax7 transcription factor during development (Jostes et al., 1990; Stoykova and Gruss, 1994; Stuart et al., 1994; Kawakami et al., 1997; Seo et al., 1998; Holland, 1999; McCauley and Bronner-Fraser, 2002; Shin et al., 2003; Osorio et al., 2005; Morona et al., 2011; Duan et al., 2012; Joven et al., 2013b).

As development advanced, the neural tube of *Xenopus* embryos showed a progressive radial stratification recognized on the basis of the relative position of cells from the ventricle to the pial surface, and the degree of cell packing. Thus, the location of the Pax7 cells could gradually be ascribed to the ventricular, subventricular, mantle, and superficial fiber zones. In addition, the mantle zone is progressively further subdivided into deep, middle and outer sublayers, which subsequently give rise to the complex adult nuclear configuration (Morona et al., 2011). In the following sections, the major traits of the Pax7 expression pattern found in *Xenopus* are discussed from rostral to caudal brain levels in relation to data available in other species in the frame of the most recent segmental models.

### Secondary prosencephalon

Following the modified prosomeric model, the forebrain comprises the topologically rostral secondary prosencephalon and the caudal diencephalon. The secondary prosencephalon is subdivided into the telencephalon and the hypothalamus (Puelles and Rubenstein, 2003). In agreement with all previous reports in different vertebrates, Pax7 expression in the telencephalon was not observed in *Xenopus* at any stage of development. However, intense Pax7 expression in the paraphysis of the urodele amphibian *Pleurodeles waltl* has been reported, representing from the embryonic stages a small invagination of the telencephalic roof plate, and the expression was maintained through development (Joven et al., 2013b). Similar results were not consistently obtained in *Xenopus*, most likely due to the fact that the membranous formations of the choroid plexus and the adhered paraphysis were removed when the brains were dissected out. Interestingly Pax7 expression was described in the paraphysis during development in the chick (Nomura et al., 1998). The paraphysis was described within the telencephalon because it is located rostral to the velum transversum, which classically marks its dorsal limit with the diencephalon (Warren, 1905), and according to its development it was considered a posterior telencephalic organ adjacent to the choroid plexus of the third ventricle (Kappers, 1950). Therefore, the only telencephalic structure that might express Pax7, at least in some vertebrates, would be the paraphysis.

The current synthetic neuromeric model distinguishes alar and basal parts in the hypothalamus (Puelles and Rubenstein, 2003). The alar portion of the hypothalamus is continuous with the alar portion of the diencephalon and is constituted by the supraoptoparaventricular and the suprachiasmatic regions. In the basal part of the hypothalamus, tuberal and mammillary regions are recognized (for review see Medina, 2008; Moreno and González, 2011; Puelles et al., 2012a). In *Xenopus*, Pax7 cells in the hypothalamus are restricted to the small group observed in the mammillary region, unequivocally distinguished from stage 45. The spatiotemporal sequence of appearance of these cells suggests that they are originated from the adjacent Pax7 cell population in the basal diencephalic p3 (Domínguez et al., 2013b). These cells were close to the mammillary TH cell group (Milán and Puelles, 2000) but actual colocalization of Pax7 and TH was not observed and a direct role of Pax7 in the formation of these neurons in *Xenopus* cannot be assessed, in line with results recently obtained in the urodele amphibian *Pleurodeles waltl* (Joven et al., 2013b).

Another interesting shared feature between *Xenopus* and other vertebrates is the intense expression of Pax7 in cells of the anlagen of the hypophysis from embryonic stages that in later stages was established in the intermediate lobe. The identification of this developing part of the hypophysis as the intermediate lobe was corroborated, at later stages, with double labeling for Pax7 and TH or NPY, since catecholaminergic and NPY fibers in *Xenopus* only innervate this part of the hypophysis (González et al., 1993; Tuinhof et al., 1994). Actually, studies in zebrafish and mammals have shown that Pax7 has a crucial role in the formation of the *pars intermedia* or the intermediate lobe (Guner et al., 2008; Hosoyama et al., 2010). Particularly in mammals, Pax7 constitutes a specific marker of a postnatal progenitor cell subpopulation and the melanotrope cells of the intermediate lobe of the hypophysis (Hosoyama et al., 2010; Budry et al., 2011). The cells in the intermediate lobe of *Xenopus* represent melanotrope cells that control the process of background adaptation (Tuinhof et al., 1993), and their intense Pax7 expression throughout development suggests that this transcription factor is required to accomplish such an important functional role.

### Diencephalon

According to the prosomeric model, the diencephalon comprises three segmental units: prosomeres p3, p2, and p1, from rostral to caudal, which are clearly defined by gene expression patterns and several landmarks (Puelles and Rubenstein, 2003). The p3–p1 units comprise the following major dorsal regions, conventionally conceived as extending across the alar and roof plate of the diencephalon: the prethalamus and prethalamic eminence (p3), the thalamus and epithalamus (habenula and pineal gland; p2), and the pretectum (p1). A choroid tela characterizes the whole roof of p3 and a large anterior part of the p2 roof, rostral to the habenular commissure and the pineal gland, whereas the roof of p1 distinctively shows the posterior commissure and the subcommissural organ (Puelles et al., 2012b; Puelles and Martínez, 2013).

#### P3

Conspicuous Pax7 expressing cells located in the basal part of p3 were clearly detectable in *Xenopus* from the embryonic period. Coexpression of Nkx2.1 in Pax7 cells of p3 suggests their actual basal nature, in contrast to the adjacent hypothalamus where only Nkx2.1 is expressed (Domínguez et al., 2013b). This observation is in concordance with those obtained by means of similar labeling approaches in urodele amphibians (Joven et al., 2013b) and other vertebrates (Moreno et al., 2012b). As already mentioned, the scattered Pax7 cells observed in the mammillary region, analyzed throughout development, seem to arise in the basal part of p3 and could have down regulated the expression of Nkx2.1, as it occurs in the cells originated in the medial ganglionic eminence that migrate to the cortex (Nóbrega-Pereira et al., 2010). Although actual migration was not assessed in our experiments, similar origin in the basal part of p3 for the Pax7 cells located in the hypothalamus has been proposed for amphibians and chelonians (Moreno et al., 2012b; Domínguez et al., 2013b; Joven et al., 2013b). Interestingly, in mammals the subthalamic nucleus is currently regarded as a dorsally migrated hypothalamic cells mass originated from the retromammillary area, therefore belonging to the hypothalamus (Puelles et al., 2012a). However, it was described in birds that the basal plate of p3 generates the retromammillary tegmentum and the subthalamic nucleus (García-López et al., 2008). Comparatively, the pattern of Pax7 (and Nkx2.1) expression in *Xenopus* in which Pax7 cells of the basal p3 seem to invade the mammillary region is of interest because the subthalamic nucleus of mammals (in the hypothalamus) was identified on the basis on the Pax7 expression (Stoykova and Gruss, 1994).

#### P2

Pax7 is early expressed along the vz of the roof plate, next to the choroid plexus of the epiphyseal roof. Pax7 expression in the roof plate of p2 is a characteristic also observed in chicken (Ferran et al., 2007, 2009) and mouse (Ferran et al., 2008). This is in line with the functions in the early dorsal polarization of the neural tube by Pax7, before being restricted to the roof plate of more caudal regions (Jostes et al., 1990) where the refinement of Pax7 expression participates in the regional identity of distinct nuclei (Thompson and Ziman, 2011). In contrast to the conspicuous Pax7 expression found in p3 (basal) and p1 (alar), the thalamus and the basal portion of p2 can be defined throughout development by its virtual lack of Pax expression, like in mammals (Stoykova et al., 1996; Grindley et al., 1997; Warren and Price, 1997; Kawano et al., 1999; Pratt et al., 2000; Puelles and Rubenstein, 2003).

#### P1

The extent of the caudal prosomere is marked by the retroflex fascicle that courses along the boundary between p2 and p1, whereas the caudal boundary with the mesencephalon is defined dorsally by the posterior commissure. Along the roof plate of p1 develops the subcommissural organ and multiple pretectal nuclei develop in *Xenopus* as derivatives of the alar plate (Morona et al., 2011). The alar p1 has been shown to share strikingly conserved patterns of gene expression during development in different vertebrates, which has served to the identification of three anteroposterior subdivisions of the pretectum: the precommissural (PcP), juxtacommissural, (JcP) and commissural (CoP) domains (Ferran et al., 2007, 2008, 2009; Morona and González, 2008; Merchán et al., 2011; Morona et al., 2011).

Early in *Xenopus* development, Pax7 is expressed weakly in the vz of the subcommissural organ and in the CoP domain, whereas in the latter domain it is intensely expressed in the svz and mz, in line with previous descriptions in the chicken and mouse (Ferran et al., 2007, 2009). Shortly after its first expression, many Pax7 cells also occupy the svz of the JcP domain (defined by the expression of Six3), whereas the PcP domain (defined by the expression of Ebf1, Pax3, and Xiro1) was virtually free of Pax7 cells (Morona et al., 2011). Strictly similar results were obtained in urodele amphibians during development (Joven et al., 2013b), which is in contrast to the situation in chicken and mouse where no Pax7 expression was found in the JcP domain throughout development (Ferran et al., 2007, 2008).

In general, the expression of Pax7 in the pretectal region throughout development is a conserved feature in vertebrates, including agnathans (Osorio et al., 2005), elasmobranches (Ferreiro-Galve et al., 2008), teleosts (Krauss et al., 1991; Macdonald et al., 1994; Kage et al., 2004), amphibians (Morona et al., 2011; Joven et al., 2013b), reptiles (Pritz and Ruan, 2009), birds (Ferran et al., 2007; Merchán et al., 2011), and mammals (Walther and Gruss, 1991; Stoykova and Gruss, 1994; Gerard et al., 1995; Vue et al., 2007; Ferran et al., 2008). This surely reflects the importance of Pax7 on the formation of this part of the brain, starting in the dorsal fate of the neural tube (Jostes et al., 1990) and then in the distinction of the different subdomains. Interestingly, Pax7 is regulated by Shh and, in particular, is down-regulated by Pax6 (Matsunaga et al., 2000) to establish the diencephalo-mesencephalic boundary (Puschel et al., 1992; Warren and Price, 1997; Araki and Nakamura, 1999; Lim and Golden, 2007). Actually, this boundary is formed exactly where Pax7 down-regulates its expression (Ferran et al., 2008, 2009; Morona et al., 2011; Joven et al., 2013a,b). Thus, it is possible a distinct role for Pax7 in the pretectum where it overlaps with the expression of Pax6, and in the alar mesencephalon that expresses Pax7 heavily but not Pax6.

### Mesencephalon

Our results corroborate previous studies in *Xenopus* in which abundant Pax7 expression was observed in tectal cells early in development (Chen et al., 2006; Morona et al., 2011). During the embryonic development, the mesencephalic neuroepithelium expresses Pax7 in broad domains, in both the rostral and caudal mesencephalic poles. As development proceeds, the Pax7 expression continues in elongated cells of the vz in the caudal pole and is gradually weaker toward the rostral pole in more rounded cells. Similar caudo-rostral gradients exist in embryonic mouse superior colliculus and chick tectum and Pax7 was involved in establishing tectal polarity (Thomas et al., 2004, 2007; Thompson et al., 2008) and the retino-tectal topography (Thomas et al., 2006). Actually, it was demonstrated in mice that the expression of Pax7 is upregulated during retinal innervation and axonal arborization in the tectum (Thompson et al., 2007) and, in *Xenopus*, the appearance of Pax7 expression in the tectum can also be correlated with the arrival of retinal axons, as observed with calretinin immunoreactivity (Morona and González, 2013). In addition, Pax7 and its paralogous gene Pax3 were reported to contribute to the mid-hindbrain boundary organizer maintenance (Matsunaga et al., 2000; Maczkowiak et al., 2010), preventing the posterior expansion of forebrain and midbrain fates (Matsunaga et al., 2001; Maczkowiak et al., 2010).

The lack of tegmental Pax7 expression in the mesencephalon most likely reflects the suppressive action of Shh downstream of Pax7 (Watanabe and Nakamura, 2000; Nakamura, 2001). In the case of the basal regions in p3, the action of Shh might be counteracted by the observed expression of Nkx2.1.

### Hindbrain

The hindbrain or rhombencephalon comprises the isthmic segment, frequently named rhombomere 0 (r0) and the rhombomeres 1–8 (r1–8) whose boundaries in developing *Xenopus* have been considered mainly on the basis of the localization of the motor nuclei and other ancillary markers during development (López et al., 2002; Morona and González, 2013).

During the embryonic and larval development, the dorsal alar plate (excluding the roof plate) expresses Pax7 in the mitotic active vz along the entire rhombencephalon of *Xenopus*, as described previously in other vertebrates (Jostes et al., 1990; Goulding et al., 1991; Joven et al., 2013b). The observed expression in *Xenopus* is mainly located in the ventral part of the alar plate. Pax7 labeling is first seen in the alar vz of r1 and rapidly extends caudally. This includes the Pax7 expression in the vz of the developing cerebellum, which originates from the alar part (rhombic lip region) of r1 (Aroca and Puelles, 2005; Watson, 2012). Although in a previous study on the Pax6 expression in *Xenopus* during development only the forebrain was described (Moreno et al., 2008b), the comparison of Pax7 and Pax6 expressions in the rhombic lip (own observations) suggests coexpression, as it was also observed in urodeles (Joven et al., 2013b); therefore, this region might be considered the rhombic lip of the cerebellar plate, as described in zebrafish (Wullimann et al., 2011).

The isthmus (r0) was early identified in *Xenopus* by the presence of the trochlear nucleus, ventrally, and the isthmic nucleus, dorsally (López et al., 2002). During early development, different members of the Pax gene family, such as Pax2/5/8, have been shown to be critically involved in the formation of the isthmus (Wada et al., 1998; Fritzsch and Glover, 2006), but r0 is virtually devoid of Pax7 expression in amphibians (Joven et al., 2013a,b present results). In *Xenopus*, only at the last larval stages some Pax7 labeled cells could be seen in the posterodorsal isthmic nucleus. Of note, it was demonstrated in chick embryos that Pax3 and Pax7 expressions are downregulated at the isthmus by Fgf8 and En2/Pax2–5, defining a gap between the conspicuous expression in the alar parts of the mesencephalon and r1 (Matsunaga et al., 2001).

From early stages in *Xenopus* development, a conspicuous Pax7 cell population is widely distributed in the rostral alar part of r1 (r1r), as in urodeles, birds and mammals (Aroca and Puelles, 2005; Watson, 2012; Joven et al., 2013b). From the vz of the alar r1r some Pax7 cells are progressively observed in more basal territories, intermingling with Otp cells. This observation can be readily compared to that reported in chicken where the interpeduncular nucleus arises from at least four alar and basal progenitor domains within r1 and r0 territories, characterized by differential expression of the transcription factors Pax7, Otp, Nkx6.1, and Otx2 (Lorente-Cánovas et al., 2012). Cells from each domain follow specific routes of migration to reach the interpeduncular nucleus approximately at the same stage. In particular, Pax7 cells selectively migrate out from the alar r1 and enter tangentially the adjacent basal plate (Aroca et al., 2006) in line with our observations in *Xenopus*, supporting a conserved histogenetic origin and organization of the interpeduncular nucleus. In *Xenopus*, the definitive configuration of this nucleus seems to be reached before the onset of feeding and after the retroflex fascicle reaches the interpeduncular neuropil (López et al., 2002; Morona and González, 2013), as was also observed in the urodele *Pleurodeles waltl* (Joven et al., 2012, 2013b).

### Spinal cord

In the embryonic *Xenopus* spinal cord, Pax7 is expressed in the vz of the alar plate and during the larval stages the expression is gradually reduced. In line with this observation, Pax7 has been related to the specification of the different progenitor cell identities in similar locations of the dorsal spinal cord of all vertebrates studied (Ericson et al., 1997; Diez del Corral et al., 2003; Maczkowiak et al., 2010; Karus et al., 2011; Kuscha et al., 2012). In contrast to mammals, amphibians, such as adult urodeles and anuran larvae (for example, *Xenopus*) can regenerate their spinal cord after injury (Slack et al., 2008; Tanaka and Ferretti, 2009). The expression of Pax7 in the developing and adult spinal cord in the axolot (urodele amphibian, *Ambystoma mexicanum*) has been related to the capacity of spinal regeneration (Schnapp and Tanaka, 2005; McHedlishvili et al., 2007, 2012). In contrast, in another urodele, the newt *Pleurodeles waltl*, the lack of Pax7 cells in the vz of the spinal cord in the adult has been related to the lack of regeneration (Joven et al., 2013a). In *Xenopus* tadpoles, the tail regeneration occurs through the activation of tissue-specific stem and/or progenitor cells, and it has been demonstrated that Pax7 positive satellite cells are required for skeletal muscle regeneration, resembling cellular mechanisms involved in homeostatic and reparative regeneration in mammals (Chen et al., 2006). However, while spinal cord regeneration in *Xenopus* tadpoles has been demonstrated to proceed through activation of several genes, such as Sox2, in the vz (Gaete et al., 2012), no data are available about a possible role for Pax7. Interestingly, after spinal cord transection *Xenopus laevis* can re-establish nerve tracts and achieve functional recovery but this ability is restricted to the larval stages and is lost at the end of metamorphosis (Forehand and Farel, 1982; Beattie et al., 1990; Gibbs et al., 2011), when the expression of Pax7 clearly diminishes.

## Concluding remarks

The comparative study of the expression pattern of Pax7 in the developing CNS of *Xenopus* and those described for other vertebrates has shown that they are remarkably conserved. The comparison has been readily made using the neuromeric model because the expression of Pax7 in the brain of *Xenopus* mostly respects the anatomical boundaries described in the current segmental model of organization of the brain. We have observed that Pax7 is first expressed in restricted regions in the vz of the CNS and, as development proceeds, the expression extends from these mitotic germinal zones to migrated cell groups. Such changes have led to suggest different roles for this Pax molecule, and other members of the gene Pax family, in regionalization and subdivision of the nervous system during early stages, and the differentiation of specific cell populations during late stages (Kawakami et al., 1997; Hsieh and Yang, 2009; Sansom et al., 2009). Moreover, the spatiotemporal sequence of Pax7 expression provided indirect evidences of possible migratory routes from the basal diencephalon to the mammillary hypothalamus and from alar to basal territories in the hindbrain, although further experimental studies are needed to clarify these migratory events.

## Author contributions

All authors had full access to all the data in the study and take responsibility for the integrity of the data and the accuracy of the data analysis. Agustín González and Ruth Morona devised the study. Sandra Bandín and to a lesser extent Nerea Moreno, performed all of the experiments. Sandra Bandín and Ruth Morona were the primary contributors to the data analysis. Sandra Bandín and Ruth Morona led the figure preparation and wrote the majority of the article, further completed and edited by Nerea Moreno and Agustín González All authors approved the article.

### Conflict of interest statement

The authors declare that the research was conducted in the absence of any commercial or financial relationships that could be construed as a potential conflict of interest.

## Abbreviations

ABB: alar basal boundary
al: anterior lobe of the hypophysis
aol: area octavolateralis
ap: alar plate
APt: anterior pretectal nucleus
bp: basal plate
C: central thalamic nucleus
Cb: cerebellum
cc: central canal
CoP: commissural pretectum
dh: dorsal horn of the spinal cord
Gc: griseum central
GT: griseum tectale
H: hypothalamus
il: intermediate lobe of the hypophysis
Ip: interpeduncular nucleus
ISc: inner nucleus of the subcommissural organ
JcP: juxtacommissural pretectum
LDT: laterodorsal tegmental nucleus
LPD: lateral posterodorsal nucleus
Ma: mammillary region
Mes: mesencephalon
mz: mantle zone
nl: neural lobe of the hypophysis
OPT: olivary pretectal nucleus
OSc: outer nucleus of the subcommissural organ
OT: optic tectum
p1–p3: prosomeres 1–3
PcP: precommissural pretectum
PcPC: parvocellular nucleus of the posterior commissure
Pdi: posterodorsal tegmental nucleus, isthmic part
PO: preoptic area
PrPt: principal pretectal nucleus
r0: isthmus (rhombomere r0)
r1–r8: rhombomeres 1–8
r1c: caudal part of rhombomeres 1
r1r: rostral part of rhombomeres 1
Rh: rhombencephalon
RM: retromammillary nucleus
Rm: nucleus reticularis medius
Rs: nucleus reticularis superior
sc: spinal cord
SC: suprachiasmatic nucleus
ScO: subcommissural organ
SM: superficial mammillary nucleus
SpL: spiriformis lateralis
svz: subventricular zone
Teg: mesencephalic tegmentum
Tel: telencephalon
Tmg: magnocellular nucleus of the torus semicircularis
Ts: torus semicircularis
Tub: tuberal hypothalamic region
v: ventricle
vh: ventral horn of the spinal cord
VJc: ventral juxtacommissural nucleus
vz: ventricular zone.
